# Low-Dose of Intrapulmonary Pirfenidone Improves Human Transforming Growth Factorβ1-Driven Lung Fibrosis

**DOI:** 10.3389/fphar.2020.593620

**Published:** 2020-11-27

**Authors:** Tomohito Okano, Tetsu Kobayashi, Taro Yasuma, Corina N. D’Alessandro-Gabazza, Masaaki Toda, Hajime Fujimoto, Hiroki Nakahara, Yuko Okano, Atsuro Takeshita, Kota Nishihama, Haruko Saiki, Atsushi Tomaru, Valeria Fridman D’Alessandro, Satoru Ishida, Hiromi Sugimoto, Yoshiyuki Takei, Esteban C. Gabazza

**Affiliations:** ^1^ Department of Pulmonary and Critical Care Medicine, Mie University Faculty and Graduate School of Medicine, Tsu, Japan; ^2^ Department of Immunology, Mie University Faculty and Graduate School of Medicine, Tsu, Japan; ^3^ Center for Intractable Diseases, Mie University Faculty and Graduate School of Medicine, Tsu, Japan; ^4^ Department of Diabetes, Metabolism, and Endocrinology, Mie University Faculty and Graduate School of Medicine, Tsu, Japan; ^5^ Shionogi & Co, Ltd., Osaka, Japan

**Keywords:** idiopathic pulmonary fibrosis, pirfenidone, intrapulmonary delivery, oral therapy, adverse effects, drug delivery

## Abstract

Idiopathic pulmonary fibrosis is a chronic, progressive, and lethal lung disease of unknown etiology. Antifibrotic drugs, including pirfenidone, are currently used for the treatment of the disease. The oral administration of pirfenidone is an effective therapy, as demonstrated by several clinical trials, although it causes severe adverse events in some patients. We hypothesized that low-dose intrapulmonary delivery of pirfenidone is effective in human transforming growth factorβ1-driven pulmonary fibrosis. To demonstrate our hypothesis, we compared the therapeutic efficacy of varying doses of pirfenidone administered by oral and intranasal routes in a human transforming growth factor-β1 transgenic mouse with established pulmonary fibrosis. We found similar amelioration of lung cell infiltration, inflammatory and fibrotic cytokines, lung fibrosis score, and hydroxyproline content in mice with human transforming growth factor-β1-mediated pulmonary fibrosis treated with low-dose intranasal pirfenidone and high-dose oral pirfenidone. This study showed that pirfenidone is a potent inhibitor of human transforming growth factor-β1-driven lung fibrosis and that intrapulmonary delivery of low-dose pirfenidone produces therapeutic responses equivalent to high-dose of oral pirfenidone.

## Introduction

Idiopathic pulmonary fibrosis (IPF) is a chronic, progressive, and devastating lung disease of unknown etiology that increases global morbidity and mortality (King et al., 2011; Lederer and Martinez, 2018). The life expectancy of patients with idiopathic pulmonary fibrosis is only 2–3 years after a confirmed diagnosis of the disease (Barratt et al., 2018). IPF is the most frequent form of fibrotic lung disease (Lederer and Martinez, 2018). According to recent epidemiological studies, there are more than five million cases of IPF globally, and the number of patients keeps increasing (Meltzer and Noble, 2008). Once the disease is triggered, a myriad of pro-fibrotic factors including transforming growth factor (TGF) β1, connective tissue growth factor (CTGF), inflammatory cytokines including interleukin-13 (IL-13), interferon (IFN)γ and clotting factors may contribute to disease progression and outcome (King et al., 2011; Barratt et al., 2018). However, TGFβ1 is the main driving factor of the aberrant tissue healing process in IPF. TGFβ1 may promote extracellular matrix deposition by directly stimulating the secretion of collagen, tenascin, fibronectin, inhibiting the degradative activity of metalloproteinases and stimulating the secretion of proliferating chemotactic factors of fibroblast or by promoting epithelial-mesenchymal transition (Drumm et al., 2005; Wolters et al., 2014; Rockey et al., 2015; Aschner and Downey, 2016; Richeldi et al., 2017; Caja et al., 2018). The development of severe pulmonary fibrosis resembling the human disease in mice with lung-specific overexpression of human TGFβ1 is the proof-of-concept of the pivotal role of TGFβ1 in pulmonary fibrosis (D'Alessandro-Gabazza et al., 2012; Fujiwara et al., 2017; D'Alessandro-Gabazza et al., 2018).

Pirfenidone (5-methyl-1-phenyl-2-[1H]-pyridone) (PFD) is a pyridone compound currently used as an oral antifibrotic drug for IPF therapy (King et al., 2014b). Previous studies have demonstrated that PFD exerts anti-oxidant, anti-inflammatory, and antifibrotic activity, although the precise mechanism of its beneficial effect in IPF remains unclear (Macias-Barragan et al., 2010; Lopez-de la Mora et al., 2015). Japan has approved the clinical use of oral PFD in 2008, Europe in 2011, and the United States of America in 2014 for IPF based on clinical trials' outcomes (Taniguchi et al., 2010; Noble et al., 2011; King et al., 2014a; Raghu et al., 2015). Pooled analysis of previous clinical trials showed that PFD significantly suppresses the decline of forced vital capacity, prolongs the progression-free survival, and reduces both all-cause mortality and the risk of hospitalizations (Noble et al., 2016). PFD also reduces the frequency of acute exacerbations in IPF (Iwata et al., 2016). PFD’s recommended daily dose in Europe and the United States of America is 2,403 mg/day and 1800 mg/day in Japan (Lyseng-Williamson, 2018; Richeldi et al., 2018). The patients well tolerate this daily dose of PFD, although adverse effects occurred in all of them (Taniguchi et al., 2010). Of particular clinical concern is gastrointestinal symptoms, which are frequent in Western patients (36%), and skin rash due to photosensitivity, which is more frequent in the Asian population (51%) (Taniguchi et al., 2010; King et al., 2014a; Costabel et al., 2017; Lancaster et al., 2017). Adverse events worsen the quality of life of the patients. Therefore, there is an imperative need to implement therapeutic strategies to attenuate PFD’s adverse effects in IPF. Intrapulmonary drug delivery is an excellent strategy to reduce systemic side effects, provided that the therapeutic efficacy is the same or better (Bayat and Cook, 2004).

In the present study, we evaluated the pharmacokinetics and the inhibitory effect of PFD on human TGFβ1-driven lung fibrosis and compared its therapeutic efficacy by intrapulmonary and oral administration using transgenic mice having established lung fibrosis caused by lung-specific overexpression of the full-length human TGFβ1 gene.

## Materials and Methods

### Reagents

PFD (lot No. PIRFA00502) was provided by Shionogi & Co., Ltd (Osaka, Japan). Before using it in the experiment, the drug was stored at room temperature in a tight and light-resistant container. For oral administration, pirfenidone was dissolved in 0.5% methylcellulose (MC, lot No. TWG7044) (Fujifilm Wako Pure Chemical Corporation, Osaka, Japan) and stored at 4°C until use. For intranasal administration, PFD was dissolved in physiological saline (lot No K7J80; Otsuka Pharmaceutical Factory, Inc., Tokushima, Japan) and stored at room temperature until use.

### Animals

We have previously characterized the transgenic (TG) mouse that expresses the full-length of human TGFβ1 gene (D'Alessandro-Gabazza et al., 2012; D'Alessandro-Gabazza et al., 2018). We generated this model using C57BL/6 mice by placing the human TGFβ1 one gene under the mouse surfactant protein C (SP-C) promoter using bacterial artificial chromosome (D'Alessandro-Gabazza et al., 2012). The transgenic mouse overexpresses the human TGFβ1 transgene specifically in the lung and has a high total and active human TGFβ1 in the systemic circulation and the lungs. The precise mechanism of TGFβ1 activation in this lung fibrosis mouse model is unclear; however, indirect evidence suggests that the lung microbiome plays a role in TGFβ1 activation in the model (D'Alessandro-Gabazza et al., 2020).

The TG mouse spontaneously develops progressive pulmonary fibrosis from the sixth week of age. (D'Alessandro-Gabazza et al., 2020). Transgenic founders and germline transmission of the bacterial artificial chromosome TG were assessed by genotyping (tail tissue PCR) and by measuring the blood levels of TGFβ1.

Male mice aged between 8 and 12 weeks (25–29 g of weight) were used in the experiments. Wild type (WT) male mice with the same age and weight from Japan SLC, Co. were used as controls. The mice were housed at the Experimental Animal Center of Mie University in a specific pathogen-free environment at a temperature of 20–26°C, relative humidity of 40–70%, and a constant 12-h light/12-h dark cycle. The animals were reared in plastic cages (W170 mm × L270 mm × H130 mm, CLEA Japan, Inc.) and had access to standard chow (CE-7 pellet, CLEA Japan Inc.) and tap water ad libitum.

### CT examination

Computed tomography (CT) was performed 10 days before (day 0) and after (day 22) drug administration under anesthesia using 3% isoflurane as previously described (Fujiwara et al., 2017). CT findings were scored by nine specialists in respiratory disease using a scoring system, as previously described (D'Alessandro-Gabazza et al., 2020).

### Allocation of Mice Into Therapeutic Groups

The degree of lung fibrosis was assessed by CT using a scoring system (D'Alessandro-Gabazza et al., 2020). The human TGFβ1-TG mice were separated randomly with matched CT scores into four groups to assess PFD’s dose-dependent efficacy and three groups to assess PFD pharmacokinetics. Wild-type (WT) mice were also allocated in a group to use as a negative control. Supplementary Tables S1–S4 show the groups of each experimental protocol.

### Preparation of PFD Solution

PFD for oral administration was dissolved in 0.5% MC solution using a mortar and adjusted to a concentration of 30 mg/ml, which was then used to prepare PFD suspensions of 10 and 3 mg/ml in 0.5% MC solution. PFD for intranasal administration was dissolved in physiological saline to a concentration of 0.4 mg/ml, which was then used to prepare PFD solutions of 0.12 and 0.04 mg/ml in physiological saline. All solutions were aliquoted in tubes, stored in a refrigerator at 4°C, and used within 4 weeks. We aliquoted the PFD solution in a number of tubes equivalent to the total number of single doses to use up the PFD solution in a tube during each administration.

### Monitoring of Mouse Condition and Behavior

General conditions, including body surface, nutritional status, attitude, abnormal behavior, and the mice’s excrement, were monitored during the treatment period. In case of any abnormality such as food or water consumption difficulty, symptoms of anguish (self-injurious behavior, abnormal posture, breathing disorders), prolonged or rapid weight loss (20% or more in a few days), the mouse was euthanized.

### Administration of PFD

PFD (30, 100, and 300 mg/kg) was administered orally (10 ml/kg) to mice using a flexible gavage tube, and PFD (0.04, 0.12, and 0.4 mg/head) was administered by intranasal route (40 µl/head) through both noses under anesthesia with isoflurane. Mice of the control groups (WT/SAL, WT/MC, TGFβ1-TG/SAL, TGFβ1-TG/MC) received 0.5% MC by oral route or physiological saline by the intranasal route. Each PFD dose was administered twice a day from day 1 to day 21 and only in the morning on day 22 before sacrifice. The starting day of administration was designated as day 1.

### Euthanasia and Collection of Samples

On day 22, mice were sacrificed by an overdose (120 mg/kg) of intraperitoneal pentobarbital. After mouse euthanasia, blood was sampled from the jugular vein and collected in heparinized tubes. The blood samples were then stored on ice before centrifuging at 10,000 rpm for 3 min at 4°C. Plasma samples were collected in 1.5 ml tubes and stored at −80°C until analysis. For a sampling of bronchoalveolar lavage fluid (BALF), the trachea was cannulated, and the lungs were sequentially washed two times with 0.7 ml and once with 0.6 ml of physiological saline. The total number of BALF cells was counted using a nucleocounter (ChemoMetec, Allerød, Denmark). The BALF was then centrifuged at 956 g for 10 min at 4°C. The cell pellet was re-suspended and centrifuged in a cytospin for May-Grünwald-Giemsa (Merck, Darmstadt, Germany) staining and differential cell count. The BALF supernatant was stored at −80°C until used for biochemical measurements. Then, the thorax was surgically opened, and the lung vasculature was perfused with saline before resecting the lungs. The right upper lobes, middle lobes, and accessory lobes were perfused with and fixed in 10% neutral-buffered formalin. The formalin-fixed lung lobes were embedded in paraffin and prepared for hematoxylin-eosin and Masson trichrome staining. The right lower lobes were excised, frozen in liquid nitrogen, and stored at −80°C until biochemical analysis. Two-third of the right lower lobes was excised to homogenize and measure the levels of total TGFβ1 and active TGFβ1. One-third of the right lower lobes was used for gene expression analysis of mouse CTGF, interleukin (IL)-6, IL-13, monocyte chemoattractant protein (MCP)-1, IFNγ, SP-C, mouse and human TGFβ1. The left lung lobes were excised, frozen in liquid nitrogen, and then stored at −80°C until hydroxyproline content analysis. Lung tissue stained with hematoxylin-eosin was used to evaluate the Ashcroft score as previously described (Urawa et al., 2016).

### Biochemical Analysis

Total protein was measured using the bicinchoninic acid (BCA) protein colorimetric assay kit (Thermo Fisher Scientific Inc., Waltham, MA). Collagen type I was measured by enzyme immunoassay using anti-collagen type I antibody and anti-collagen type I biotin-conjugated antibody from Rockland Immunochemicals Inc. (Limerick, PA). Total TGFβ1 and active TGFβ1 were measured using enzyme immunoassay kits from R&D Systems (Minneapolis, MN), and mouse IFNγ was measured by an enzyme immunoassay kit from BD Bioscience (BD opt-EIA kits, San Diego, CA). The immunoassay for TGFβ1 shows cross-reactivity with human and mouse TGFβ1. Surfactant protein D (SP-D) was measured using a commercial enzyme-linked immunosorbent assay kit from Sino Biologicals (Beijing, China); briefly, the kit contains a monoclonal antibody specific or mouse SP-D that is coated to plate wells. The standards and samples are added to the wells, followed by incubation. After appropriate washing, a solution containing horseradish peroxidase-conjugated anti-mouse SP-D is added before color development with tetramethylbenzidine.

### Hydroxyproline Analysis

Hydroxyproline content was measured by a colorimetric method using a commercial kit (Hydroxyproline colorimetric assay kit, BioVision, San Francisco, CA) following the manufacturers' instructions.

### Gene Expression Analysis

Total RNA was extracted from the lungs using Trizol (Thermo Fisher Scientific Inc., Waltham, MA), and cDNA was synthesized using reverse transcriptase (Thermo Fisher Scientific Inc., Waltham, MA) and oligo dT. The mRNA expression of cytokines and growth factors was evaluated by PCR using primers (Supplementary Table S5), as described (Fujiwara et al., 2017). We normalized the mRNA expression by the glyceraldehyde-3-phosphate dehydrogenase transcript level.

### Determination of PFD in Plasma

The concentration of PFD in plasma was determined using high-performance liquid chromatography with tandem mass spectrometric detection (LC/MS/MS) and the data acquisition software Analyst (AB SCIEX, Framingham, MA) following validated method. Plasma values below the lower limit of quantification were not included in the analysis. Pharmacokinetic parameters including the maximum concentration (Cmax), time-to-reach the maximum concentration (Tmax), elimination half-life (t1/2,z), the area under the concentration-time curve (AUC) from zero to infinity time (AUCinf) were calculated using the post-analysis processing software Phoenix WinNonlin (Certara USA, Inc., Princeton, NJ).

### Blood Collection and Processing for Pharmacokinetic Study

Blood was collected at 5, 10, 30 min and 1, 3, and 6 h after intranasal or oral administration on day 1 and 0, 5, 10, 30 min and 1, 3, and 6 h after intranasal or oral administration on day 22. Serial blood sampling was performed through the lateral tail vein. The first blood sample (30 µl) was collected using a heparinized capillary tube after a slight incision of the lateral tail vein with an injection needle. During subsequent samplings, the incision scab was removed using absorbent cotton, the lateral tail vein is stroked gently, and blood was collected using a capillary tube via the kerf. We stopped bleeding after sampling by applying pressure to the kerf. The blood sample was then transferred to a tube and centrifuged at 10,000 rpm for 3 min at 4°C to separate plasma. After collecting blood for 6 h after administration on day 22, mice are euthanized by an intraperitoneal overdose administration of pentobarbital (120 mg/kg).

### Statistical Analysis

Data were expressed as the mean ± standard errors of the means (S.E.M.). The statistical difference between WT mice and TGFβ1-TG mice was calculated by unpaired *t*-test with Welch’s correction. The statistical difference between vehicle-treated mice and PFD treated mice was calculated by analysis of variance (ANOVA) followed by Dunnett’s *post hoc* test. Statistical analyses were performed using the Graph-pad Prism version 7.0 (Graph-pad Software, San Diego, CA). A *p* < 0.05 was considered as statistically significant.

## Results

### PFD Ameliorates Radiological Findings

**FIGURE 1:**
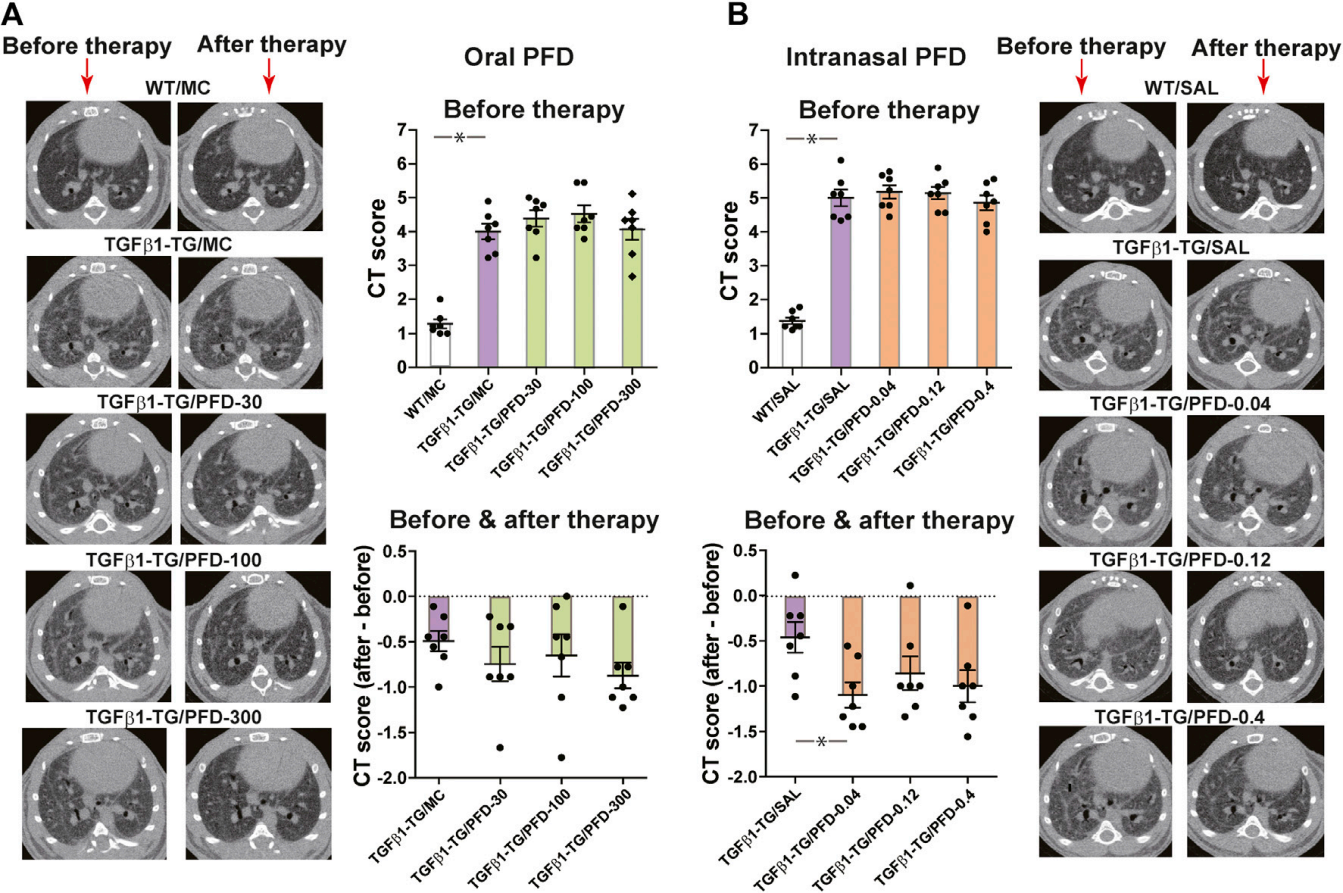
Computed tomography (CT) score of fibrosis significantly improved after treatment with a low dose of intranasal pirfenidone (PFD) compared to control. Human transforming growth factor (TGF)β1 transgenic (TG) mice were allocated in groups treated with PFD by oral (**A**; *n* = 7) or intranasal (**B**; *n* = 7) administration and in groups treated with the vehicle saline (SAL; *n* = 7) or with the vehicle methylcellulose (MC; *n* = 7) twice a day for 21 days and once a day on day 22 before euthanasia. Wild type (WT; *n* = 7) mice treated with saline or MC were used as negative controls. CT was performed as described under materials and methods. CT score of each mouse is the mean score of nine readers blinded for the treatment and mouse groups. Data are expressed as the mean ± S.E.M. Statistical analysis by Student’s *t*-test and ANOVA with Dunnett’s *post hoc* test. *p < 0.05.

The CT score in TGFβ1-TG/MC mice was significantly higher than in WT/MC mice before starting oral MC. There were no significant differences in CT scores among TGFβ1-TG groups before starting oral PFD. The CT scores decreased in all groups treated with oral PFD compared to the TGFβ1-TG/MC, although the decrease was not significant (Figure 1A).

The CT score in TGFβ1-TG/SAL mice was significantly higher than in WT/SAL mice before intranasal SAL. There were no significant differences in CT scores among TGFβ1-TG groups before intranasal PFD. The CT score significantly decreased in the TGFβ1-TG/PFD-0.04 group compared to the TGFβ1-TG/SAL group after intranasal PFD. The CT score also decreased in the TGFβ1-TG/PFD-0.12 and TGFβ1-TG/PFD-0.4 groups compared to the TGFβ1-TG/SAL group after intranasal PFD although the decrease was not significant (Figure 1B).

Because of the lack of consistency with changes observed in other variables, the modest improvement of CT scores in the TGFβ1-TG/MC TGFβ1-TG/SAL groups was probably due to variability in the CT scoring system we used in the present study.

### PFD Reduces Lung Cell Infiltration

**FIGURE 2:**
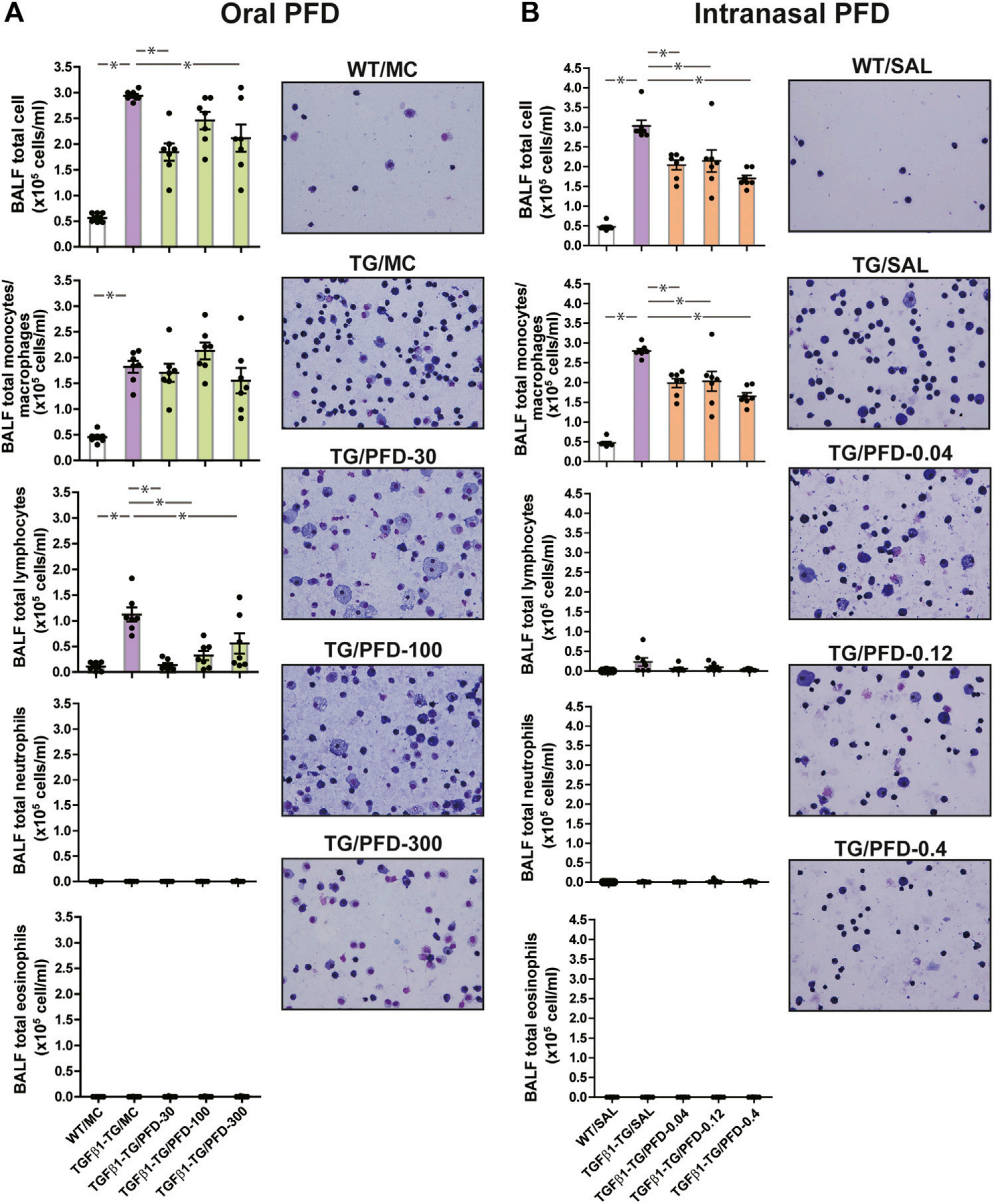
Significant reduction in the number of inflammatory cells in the lungs from mice treated with pirfenidone (PFD) by oral or intranasal administration compared to controls. Human transforming growth factor (TGF)β1 transgenic (TG) mice were allocated in groups treated with PFD by oral (**A**; *n* = 7) or intranasal (**B**; *n* = 7) administration and in groups treated with the vehicle saline (SAL; *n* = 7) or with the vehicle methylcellulose (MC; *n* = 7) twice a day for 21 days and once a day on day 22 before euthanasia. Wild type (WT; *n* = 7) mice treated with SAL or MC were used as negative controls. Bronchoalveolar lavage fluid (BALF) cells were counted using a nucleocounter and stained with Wright-Giemsa as described under materials and methods. Scale bars indicate 50 µm. Data are expressed as the mean ± S.E.M. Statistical analysis by Student’s *t*-test and ANOVA with Dunnett’s *post* hoc test. *p < 0.05.

The total count of cells, the absolute number of monocytes/macrophages, and lymphocytes in BALF were significantly increased in TGFβ1-TG/MC mice compared to WT/MC mice. The BALF total cells were significantly decreased in TGFβ1-TG/PFD-30 and TGFβ1-TG/PFD-300 groups and the absolute number of BALF lymphocytes was significantly reduced in TGFβ1-TG/PFD-30, TGFβ1-TG/PFD-100 and TGFβ1-TG/PFD-300 groups compared to the TGFβ1-TG/MC group (Figure 2A).

The total cell count and the absolute number of monocytes/macrophages in BALF were significantly increased in TGFβ1-TG/SAL mice compared to WT/SAL mice. In mice receiving intranasal PFD, the total count of BALF cells and the absolute number of BALF macrophages were significantly decreased in all PFD-treated TGFβ1-TG groups compared to the control TGFβ1-TG/SAL group (Figure 2B).

### PFD Reduces Lung IFNγ Levels

Of mice receiving MC alone by oral route or intranasal saline, the BALF IFNγ level was significantly increased in TGFβ1-TG mice compared to WT mice. In mice receiving oral or intranasal PFD, the BALF IFNγ was significantly decreased in PFD-treated TGFβ1-TG groups compared to the control TGFβ1-TG/MC or TGFβ1-TG/SAL group (Supplementary Figures S1A,B).

### PFD Reduces the mRNA Expression of Inflammatory Cytokines

**FIGURE 3:**
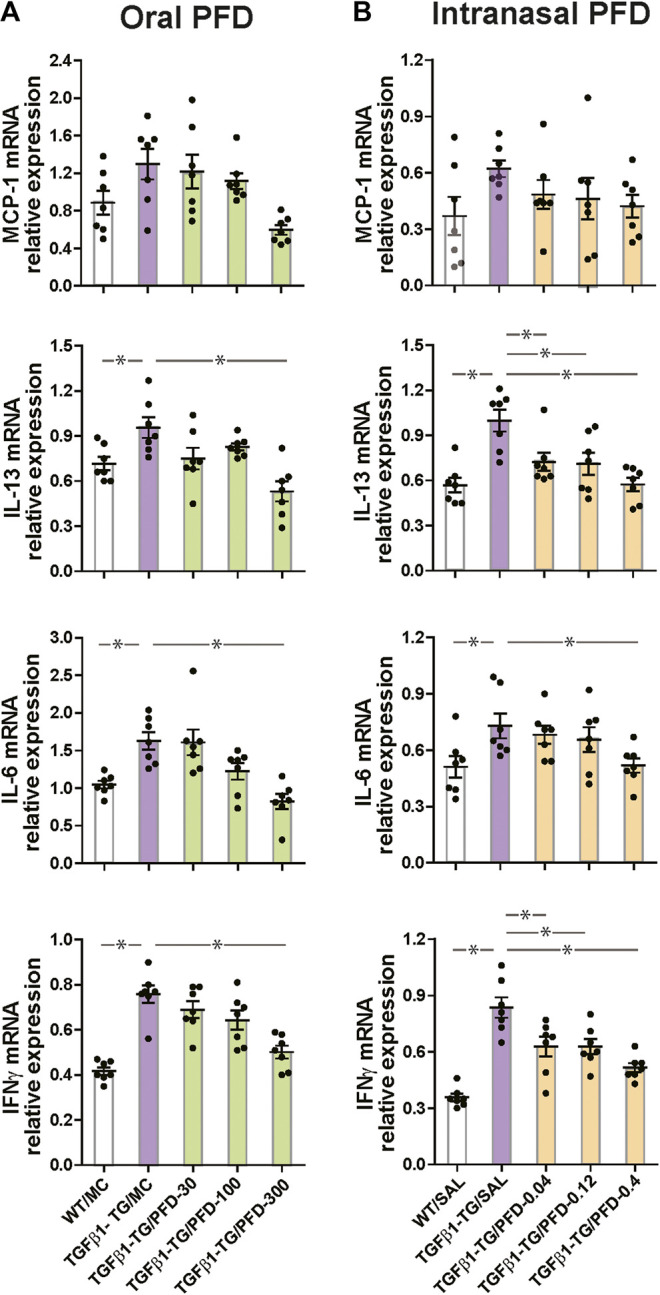
Significant reduction in the relative mRNA expression of inflammatory cytokines in the lungs from mice treated with pirfenidone (PFD) by oral or intranasal administration compared to controls. Human transforming growth factor (TGF)β1 transgenic (TG) mice were allocated in groups treated with PFD by oral (**A**; *n* = 7) or intranasal (**B**; *n* = 7) administration and in groups treated with the vehicle saline (SAL; *n* = 7) or with the vehicle methylcellulose (MC; *n* = 7) twice a day for 21 days and once a day on day 22 before euthanasia. Wild type (WT; *n* = 7) mice treated with saline or MC were used as negative controls. The relative mRNA expression of monocyte chemoattractant protein-1 (MCP-1), interleukin (IL)-13, IL-6, and interferonγ (IFNγ) was evaluated by RT-PCR as described under materials and methods. Data are expressed as the mean ± S.E.M. Statistical analysis by Student’s *t*-test and ANOVA with Dunnett’s *post hoc* test. *p < 0.05.

The relative mRNA expressions of IL-13, IL-6, and IFNγ were significantly increased in the TGFβ1-TG/MC group compared to the WT/MC group. The relative mRNA expression of MCP-1, IL-13, IL-6, and IFNγ were significantly reduced in TGFβ1-TG/PFD-300 mice compared to TGFβ1-TG/MC mice (Figure 3A).

Among mice receiving intranasal saline, there was no significant difference in the relative mRNA expression of MCP-1 among all groups. The relative mRNA expressions of IL-13, IL-6, and IFNγ were significantly increased in the TGFβ1-TG/SAL group compared to the WT/SAL group. The mRNA expressions of IL-13, IL-6, and IFNγ were significantly reduced in the TGFβ1-TG/PFD-0.4 group compared to the TGFβ1-TG/SAL group. The mRNA expression of IL-13 and IFNγ but not that of IL-6 was significantly decreased in the TGFβ1-TG/PFD-0.04 and TGFβ1-TG/PFD-0.12 groups compared to the TG/SAL group (Figure 3B).

### PFD Reduces the Expression of SP-D

The plasma concentration of SP-D was significantly increased in TGFβ1-TG/MC mice compared to WT/MC mice. However, the plasma SP-D level was significantly decreased in TGFβ1-TG/PFD-30, TGFβ1-TG/PFD-100, and TGFβ1-TG/PFD-300 groups compared to the TGFβ1-TG/MC group (Supplementary Figure S2A).

The plasma concentrations of SP-D was significantly increased in TG/SAL mice compared to WT/SAL mice. However, the plasma concentration of SP-D was significantly decreased in TGFβ1-TG/PFD-0.04 group, TGFβ1-TG/PFD-0.12, and TGFβ1-TG/PFD-0.4 groups compared to the TGFβ1-TG/SAL group (Supplementary Figure S2B).

### PFD Reduces Lung Fibrosis Score

The Ashcroft score was significantly increased in the lungs from TGFβ1-TG/MC compared to WT/MC mice. However, the Ashcroft score was significantly decreased in the TGFβ1-TG/PFD 300 group compared to the TG/MC group. The Ashcroft score in the TGFβ1-TG/PFD-30 and TGFβ1-TG/PFD-100 groups was low compared to the TG/MC group, although the improvement was not significant (Supplementary Figure S3A).

The Ashcroft score was significantly increased in TGFβ1-TG/SAL mice compared to WT/SAL mice. In mice receiving intranasal PFD, the fibrosis score was significantly decreased after treatment in the TGFβ1-TG/PFD-0.4 group compared to the TG/SAL group. The Ashcroft score in the TGFβ1-TG/PFD-0.04 and TGFβ1-TG/PFD-0.12 groups was low compared to the TG/SAL group, although the improvement was not significant (Supplementary Figure S3B).

### PFD Reduces Lung Collagen Deposition

**FIGURE 4:**
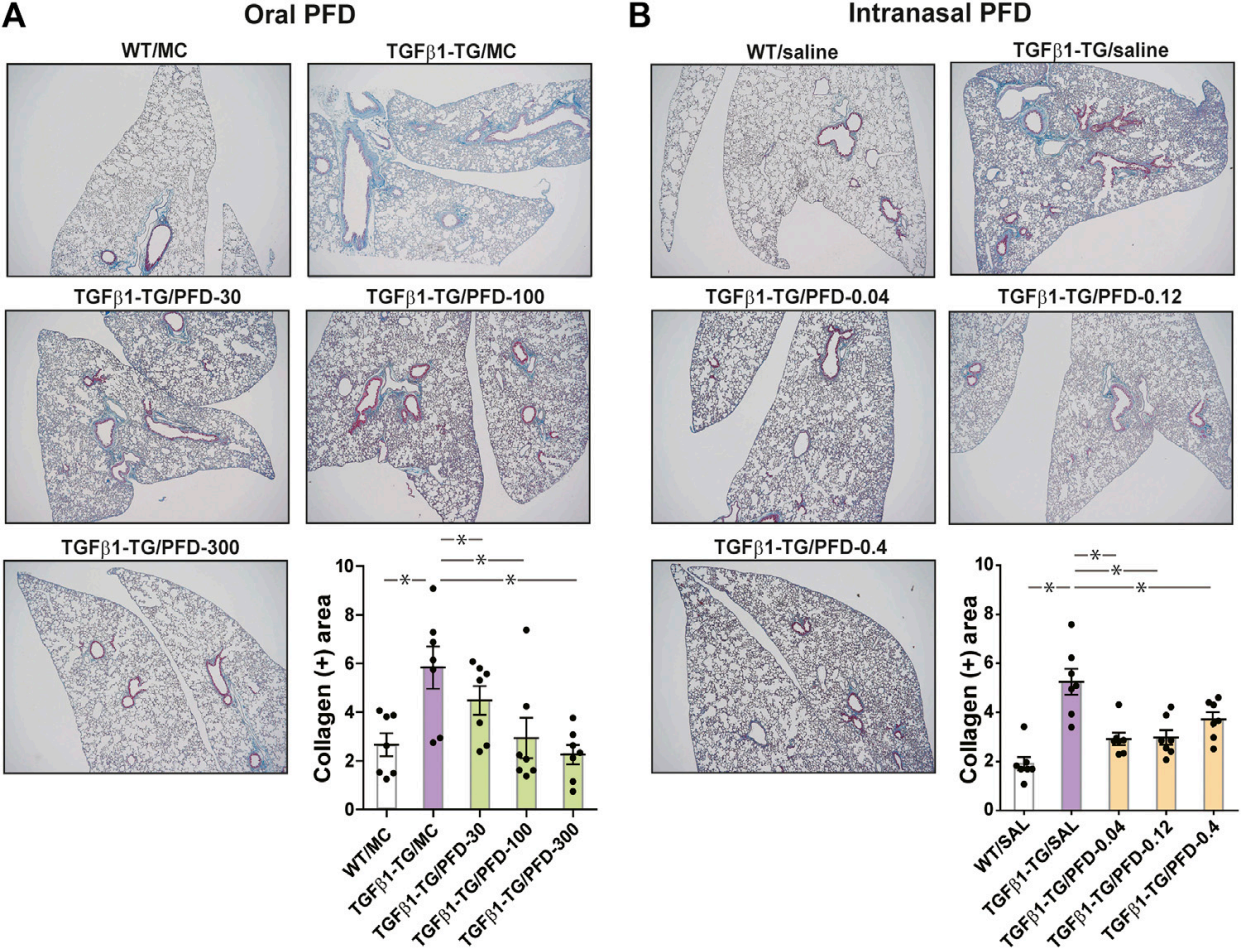
Significant reduction in the lung fibrotic area after treatment with oral or intranasal pirfenidone (PFD) compared to controls. Human transforming growth factor (TGF)β1 transgenic (TG) mice were allocated in groups treated with PFD by oral (**A**; *n* = 7) or intranasal (**B**; *n* = 7) administration and in groups treated with the vehicle saline (SAL; *n* = 7) or with the vehicle methylcellulose (MC; *n* = 7) twice a day for 21 days and once a day on day 22 before euthanasia. Wild type (WT; *n* = 7) mice treated with saline or MC were used as negative controls. Lung tissue was stained with Masson’s trichrome, and the fibrotic stained area was calculated using the WINDROOF image software as described under materials and methods. Scale bars indicate 500 µm. Data are expressed as the mean ± S.E.M. Statistical analysis by Student’s *t*-test and ANOVA with Dunnett’s *post hoc* test. *p < 0.05.

The collagen positive area was significantly increased in the lungs from TGFβ1-TG/MC or TGFβ1-TG/SAL mice compared to WT/MC or WT/SAL mice. In mice receiving oral or intranasal PFD, the collagen positive area was significantly decreased in the lungs from all PFD-treated TGFβ1-TG groups compared to the TGFβ1-TG/MC or TGFβ1-TG/SAL group (Figure 4A,B).

### PFD Reduces Lung Collagen Markers

**FIGURE 5:**
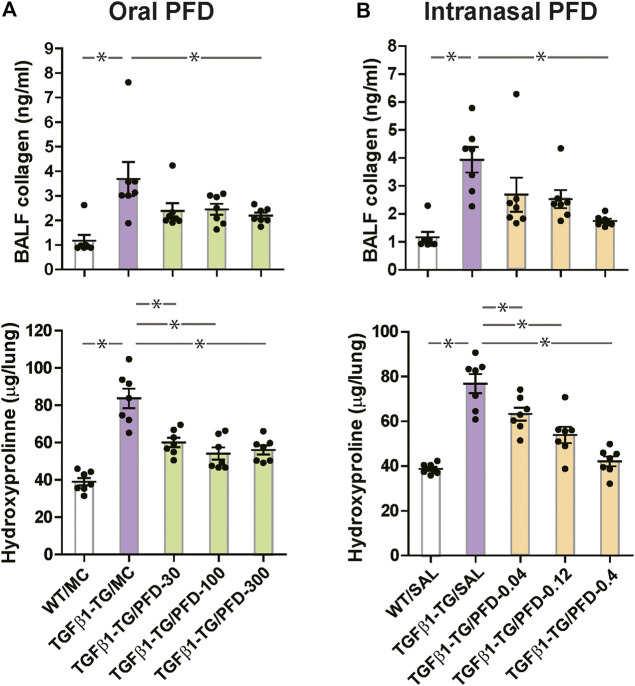
Significant reduction in collagen deposition markers in the lungs from mice treated with pirfenidone (PFD) by oral or intranasal administration compared to controls. Human transforming growth factor (TGF)β1 transgenic (TG) mice were allocated in groups treated with PFD by oral (**A**; *n* = 7) or intranasal (**B**; *n* = 7) administration and in groups treated with the vehicle saline (SAL; *n* = 7) or with the vehicle methylcellulose (MC; *n* = 7) twice a day for 21 days and once a day on day 22 before euthanasia. Wild type (WT; *n* = 7) mice treated with saline or MC were used as negative controls. Data are expressed as the mean ± S.E.M. Statistical analysis by Student’s *t*-test and ANOVA with Dunnett’s *post hoc* test. *p < 0.05.

The BALF concentration of collagen I and the lung content of hydroxyproline were significantly increased in TGFβ1-TG/MC mice compared to WT/MC mice. The BALF concentration of collagen I was significantly decreased in the TGFβ1-TG/PFD-300 group compared to the TGFβ1-TG/MC group. The lung hydroxyproline was significantly reduced in the TGFβ1-TG/PFD-30, TGFβ1-TG/PFD-100, and TGFβ1-TG/PFD-300 groups after oral PFD compared to the TGFβ1-TG/MC group (Figure 5A).

The BALF concentration of collagen I and the lung hydroxyproline were significantly increased in TGFβ1-TG/SAL mice compared to WT/SAL mice. In mice receiving intranasal PFD, the BALF concentration of collagen I was significantly decreased in the TGFβ1-TG/PFD-0.4 group compared to the TGFβ1-TG/SAL group. The lung content of hydroxyproline was significantly reduced in the TGFβ1-TG/PFD-0.04, TGFβ1-TG/PFD-0.12, and TGFβ1-TG/PFD-0.4 groups compared to the TGFβ1-TG/SAL group (Figure 5B).

### PFD Reduces the Lung Concentrations of Growth Factors

**FIGURE 6:**
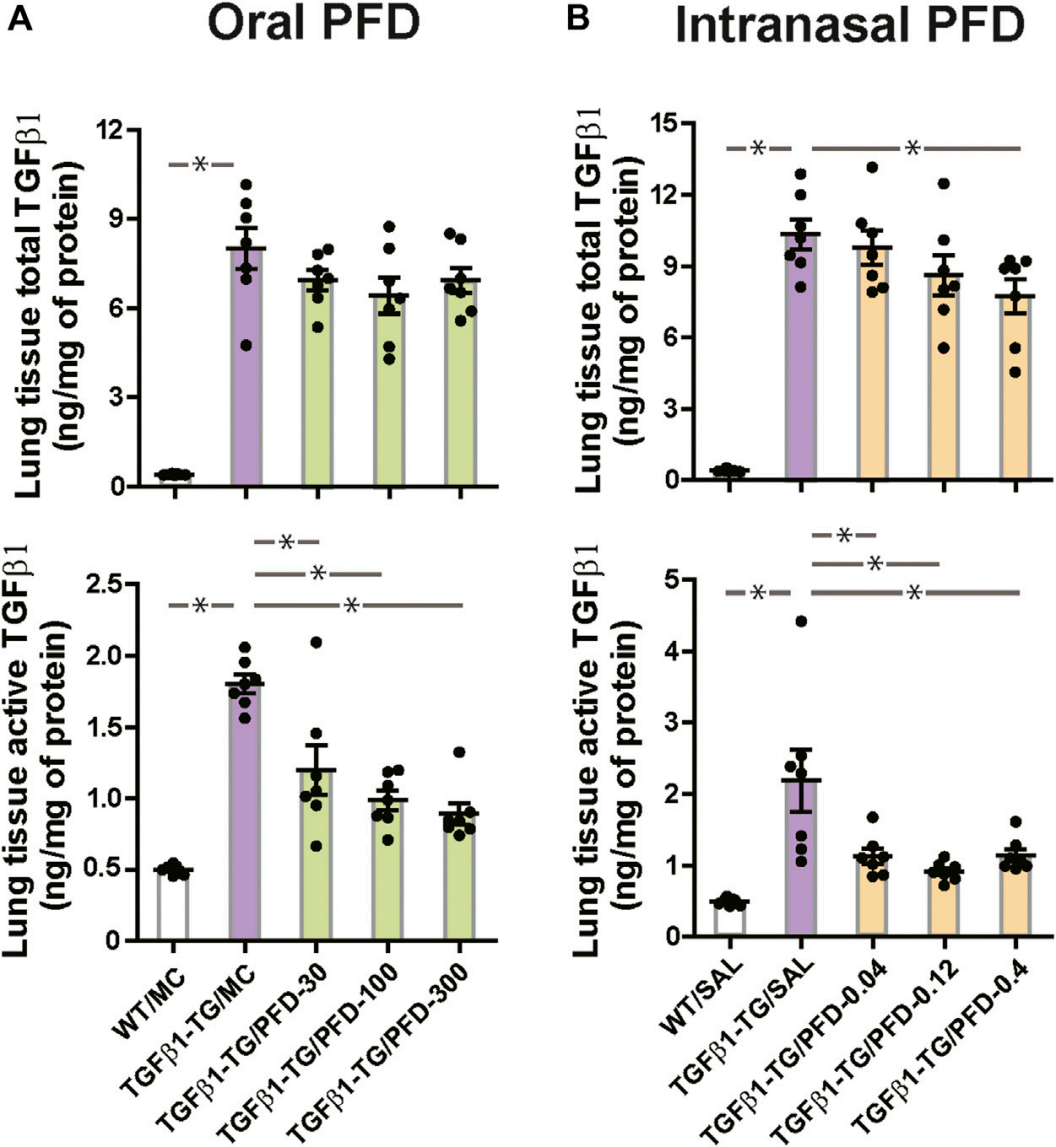
Significant reduction in the level of transforming growth factor-β1 (TGFβ1) in the lungs from mice treated with pirfenidone (PFD) by oral or intranasal administration compared to controls. Human transforming growth factor (TGF)β1 transgenic (TG) mice were allocated in groups treated with PFD by oral (**A**; *n* = 7) or intranasal (**B**; *n* = 7) administration and in groups treated with the vehicle saline (SAL; *n* = 7) or with the vehicle methylcellulose (MC; *n* = 7) twice a day for 21 days and once a day on day 22 before euthanasia. Wild type (WT; *n* = 7) mice treated with saline or MC were used as negative controls. Data are expressed as the mean ± S.E.M. Statistical analysis by Student’s *t*-test and ANOVA with Dunnett’s *post hoc* test. *p < 0.05.

The lung tissue concentration of total TGFβ1, the lung tissue concentration of active TGFβ1 (Figure 6A), the lung mRNA relative expressions of human TGFβ1, and CTGF were significantly increased in TGFβ1-TG/MC mice compared to WT/MC mice (Supplementary Figure S4A).

The lung tissue concentration of active TGFβ1 was significantly decreased in all PFD-treated TGFβ1-TG groups compared to the TGFβ1-TG/MC group (Figure 6A). The relative mRNA expression of CTGF was significantly decreased in TGFβ1-TG/PFD-100 and TGFβ1-TG/PFD-300 groups compared to the TGFβ1-TG/MC group (Supplementary Figure S4A).

In control mice, the lung tissue concentration of total TGFβ1, the lung tissue concentration of active TGFβ1 (Figure 6B), the lung relative mRNA expressions of human TGFβ1, and CTGF were significantly increased in TGFβ1-TG/SAL mice compared to WT/SAL mice (Supplementary Figure S4B). The relative mRNA expression of mouse TGFβ1 was not significantly different between TGFβ1-TG/SAL and WT/SAL groups (Supplementary Figure S4B).

In mice receiving intranasal PFD, the lung tissue total TGFβ1 was significantly decreased in the TGFβ1-TG/PFD-0.4 group, and the lung tissue active TGFβ1 was significantly decreased in all PFD-treated TGFβ1-TG groups compared to the TGFβ1-TG/SAL group (Figure 6B). The relative mRNA expression of CTGF was significantly decreased in TGFβ1-TG/PFD-0.12 and TGFβ1-TG/PFD-0.4 groups compared to TGFβ1-TG/SAL mice (Supplementary Figure S4B).

### Repeated Oral Administrations Affect Drug Pharmacokinetics

**FIGURE 7:**
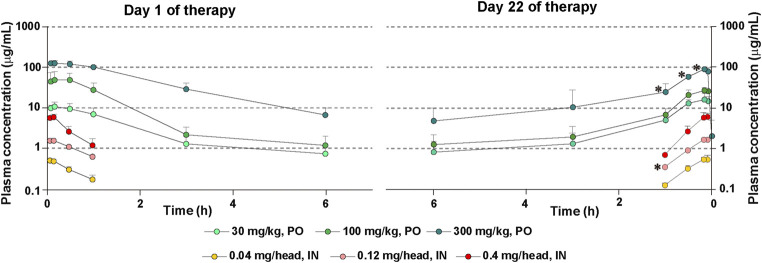
Plasma levels of PFD after repeated administrations of PFD by the oral or intranasal route. Human transforming growth factor (TGF)β1 transgenic (TG) mice were allocated in groups treated with PFD by oral (*n* = 4) or intranasal (*n* = 4) administration and blood samples were drawn from each group on day 1 and day 22. Bars indicate the mean ± S.E.M. Statistical analysis by Student’s *t*-test with Welch’s correction. *p < 0.05.

As expected, there was a dose-dependent increase in PFD’s plasma concentrations after its administration, the concentrations being higher in mice treated by oral route than in mice treated by the intranasal route (Figure 7). In mice treated with 300 mg/kg of oral PFD, the PFD concentration was significantly decreased on day 22 compared to day 1 in plasma sampled 10 min, 30 min, and 1 h after PFD administration (Figure 7).

There was no significant difference in PFD concentrations in plasma sampled on day 1 and day 22 after oral administration of 30 or 100 mg/kg of the drug. Cmax and AUCinf were significantly reduced in mice treated with 300 mg/kg of oral PFD on day 22 compared to mice receiving the same oral dose on day 1 (Supplementary Table S6). On the other hand, the concentration of PFD after intranasal administration of 0.12 mg/head of PFD on day 22 was significantly decreased compared to the drug concentration on day 1 in plasma sampled 1 h after PFD administration (Figure 7). AUCinf was significantly reduced in mice treated with 0.12 mg/head of intranasal PFD on day 22 compared to mice receiving the same dose on day 1 (Supplementary Table S6). PFD’s half-life in circulation on day 22 was shorter than that on day 1 in mice treated with intranasal PFD. However, PFD’s half-life in circulation on day 22 was longer than that on day 1 in mice treated with oral PFD (Supplementary Table S6).

## Discussion

This study compared the therapeutic efficacy between the oral and intranasal PFD administration in mice with human TGFβ1-driven lung fibrosis. We found comparable antifibrotic responses despite using several-fold higher doses of PFD in mice treated by oral delivery than in mice treated by the intranasal route.

Accumulated evidence from *in vitro* experiments and animal models of organ fibrosis has demonstrated PFD’s suppressive property on inflammation and fibrosis (Schaefer et al., 2011). Consistent with this previous evidence, here we found significant suppression of lung fibrosis (eg, collagen deposition area, Ashcroft and CT fibrosis score) and reduction of lung inflammation (lung cell inflammatory cells and cytokines) in TGFβ1-TG mice with pulmonary fibrosis treated with PFD (Macias-Barragan et al., 2010). Of mechanistic relevance is the decreased concentration of active TGFβ1 in mice treated with PFD compared to untreated mice. Previous studies have shown that PFD decreases the expression of TGFβ1 by blocking the expression of Smad proteins and activation of the TGFβ1/Smad2/3 signaling pathway, which enhances TGFβ1 transcription (Iyer et al., 1999; Choi et al., 2012; Sun et al., 2018; Lv et al., 2020). Another study has shown that TGFβ1 does not affect the activation of TGFβ1 by αVβ6 integrin (Porte and Jenkins, 2014). Therefore, less availability of TGFβ1 protein due to PFD-mediated inhibition of TGFβ1 protein secretion is the probable explanation for the decreased concentration of active TGFβ1 in mice treated with PFD.

The recommended oral dose of PFD prescribed in clinical practice is 1800 mg/day in Japan and 2,403 mg/day in Europe and United States, administered as one 600 mg or 801 mg tablet three times daily. This dose corresponds to 25.7–34.3 mg/kg/day for a patient with 70 kg of body weight (Lyseng-Williamson, 2018; Richeldi et al., 2018). In our present experimental mouse study, we administered two times a day by oral gavage a dose of 30, 100, or 300 mg/kg of PFD, of which 30 mg/kg is the approximate dose currently used in clinical practice. The Cmax obtained after single or multiple administrations of 30 mg/kg dose of oral PFD was 10.6 or 16.7 μg/ml, respectively. Similar to these, Cmax values were reported after a single (600 mg) or multiple administrations (400 mg, three times a day) of oral PFD in a healthy population (Shi et al., 2007). Here we showed that a low dose of PFD (30 mg/kg, two times a day) effectively decreased cell infiltration, inflammatory cytokines, levels of epithelial markers, the hydroxyproline content, and the concentration of active TGFβ1 in the lungs. However, doses of 200 mg/kg/day (Cmax range of 49.6–27.5 μg/ml) or 600 mg/kg/day (Cmax range of 134.0–90.4 μg/ml) were required to improve lung radiological findings and to reduce the area of collagen deposition and the score of tissue fibrosis in the lungs. These observations suggest the need to use higher doses of oral PFD in order to achieve a much optimal therapeutic response. However, the use of escalating doses of oral PFD is not currently recommended in clinical practice due to the high risk of inducing adverse events (Lancaster et al., 2017; Lyseng-Williamson, 2018).

Intrapulmonary delivery may be an alternative approach to overcome the adverse effect-related limitation for escalating PFD dose. In general, the intrapulmonary administration requires lower doses of a drug to achieve therapeutic responses, and it is associated with less frequent systemic side effects (Bayat and Cook, 2004). Here, we evaluated whether intrapulmonary delivery of PFD is therapeutically effective in TGFβ1-driven lung fibrosis. We treated TG mice with PFD twice a day by intranasal route using doses of 0.04 mg (3.7 mg/kg/day, calculated using the mouse body weight of day 1), 0.12 mg (10.7 mg/kg/day) or 0.4 mg (35.1 mg/kg/day) per mouse. These intranasal doses of PFD are approximately the same (34.3/35.1) as, or 3 (34.3/10.7) and 9 (34.3/3.7) times lower than the dose of oral PFD (34.3 mg/kg/day) currently used for IPF therapy in Western countries. Despite the low PFD dose, the response to intranasal PFD was equivalent to, or even better than, that achieved with high doses of oral PFD.

Interestingly, even low doses of intranasal PFD (3.7 mg/kg/day) per mouse were sufficient to ameliorate radiological findings, cell infiltration, levels of inflammatory (IFNγ) and pro-fibrotic cytokines (IL-13, active TGFβ1), the plasma level of SP-D and collagen deposition (hydroxyproline) in the lungs. Further improvement in lung inflammation markers and fibrosis was observed when the dose of intranasal PFD was increased to 35.1 mg/kg/day per mouse. These observations suggest the potential of intranasal PFD for improving therapeutic efficacy and for reducing the risk of adverse events during the treatment of pulmonary fibrosis. The PFD’s property of targeting alveolar type II cells may explain the therapeutic effect of intrapulmonary PFD. Previous reports demonstrated that PFD attenuates endoplasmic reticulum stress and mitochondrial dysfunction, inhibits apoptosis, and reduces extracellular matrix proteins' secretion in alveolar epithelial type II cells (Hisatomi et al., 2012; Du et al., 2020). A recent study showing the tolerability of aerosolized PFD in healthy and IPF subjects supports the safety of PFD’s intrapulmonary administration (Khoo et al., 2020).

The pharmacokinetics results observed in the present study also favor the use of PFD by the intranasal route. Thirty minutes after the first intranasal administration of PFD on day 1, the plasma concentrations of PFD were approximately 25 (low-dose), 40 (intermediate-dose), and 43 (high-dose) times lower than the plasma concentrations after its first oral administration. Similarly, after the first intranasal administration of PFD on day 1, the Cmax values were approximately 20 (low-dose), 30 (intermediate-dose), 20 (high-dose) times, and the AUCinf values were approximately 42 (low-dose), 45 (intermediate-dose), and 67 (high-dose) times lower than the Cmax and AUCinf values of PFD after its first oral administration. The elimination rate of PFD from the circulation after the first intranasal administration of PFD on day 1 was also approximately 2.6 (low-dose), 1.9 (intermediate-dose), and 2.5 (high-dose) times faster than after its first oral administration. These observations suggest a significant margin of safety in favor of intranasal PFD vs. oral PFD. In addition, in this study, we also addressed whether repeated intranasal or oral therapy with PFD affects the pharmacokinetic parameters of the drug. We found low plasma concentrations, Cmax and AUCinf values of PFD in mice receiving 300 mg/kg of oral PFD, and low AUCinf values in mice receiving 0.12 mg/head of intranasal PFD on day 22 compared to day 1. These findings suggest that PFD’s chronic administration accelerates its metabolism leading to decreased PFD availability in the circulation. PFD is metabolized in the liver mainly by CYP1A2 and to less extent by other isoenzymes (CYP2C9, CYP2C19, CYP2D6, CYP2E1), leading to the formation of 5-hydroxymethyl pirfenidone and 5-carboxylic acid metabolite (Schaefer et al., 2011). Inhibitors or inducers of these enzymes may modify the metabolism and pharmacokinetics of PFD, and thus their concomitant use may affect the biological activity of PFD *in vivo* and the occurrence of drug-related adverse events (Yu et al., 2018). In this context, a previous study suggested that PFD may increase the activity of cytochrome p450 in rats (Wu et al., 2014). Based on this evidence, we can speculate that the change in PFD pharmacokinetic on day 22 compared to day 1 observed in our present study was due to increased activity of cytochrome isoenzymes during the prolonged use of PFD.

### Limitations

This study demonstrated the therapeutic efficacy of low-dose PFD by the intrapulmonary route in lung fibrosis. However, this beneficial effect of intrapulmonary PFD will depend on fibrotic lung disease’s clinical stage. Many regions of the lungs of IPF patients in advanced stages are substantially less ventilated due to progressive tissue remodeling, alveolar collapse, and collapsed tissue induration that could limit the intrapulmonary drug delivery (Katzenstein and Myers, 1998). Another limitation of the present study is that the comparison of PFD therapeutic efficacy between the oral and intranasal drug delivery was performed only in the TGFβ1-associated lung fibrosis model. Future studies should evaluate whether similar results are observed using other models, including the bleomycin-induced lung fibrosis model.

## Conclusion

In brief, the results of this study showed for the first time that PFD is a potent inhibitor of human TGFβ1-driven lung fibrosis *in vivo* and that intrapulmonary delivery of low doses of PFD evokes therapeutic response equivalent to high doses of oral PFD. These observations provide a robust and convincing rationale for taking action to develop PFD inhalation therapy for IPF patients in clinical practice.

## Data Availability Statement

The original contributions presented in the study are included in the article/Supplementary Material, further inquiries can be directed to the corresponding author.

## Ethics Statement

The animal study protocol was approved by the Mie University Committee for Animal Care and Use (Approval number: 29-23).

## Author Contributions

ECG, TK, HF, SI, and HS contributed to the conceptualization and idea of the study. CND’-G, TY, KN, AT (9th author), and AT (12th author) prepared the mouse transgenic mice with lung fibrosis. MT, VFD’A, YO, HN, and HS measured several parameters in the mouse model. TK, HF, SI, and HS contributed to resources and supervision of data analysis. TO prepared the first draft of the manuscript. TK, ECG, and CND’-G made intellectual contributions and edited the manuscript.

## Funding

This research was supported in part by a grant from the Ministry of Education, Culture, Sports, Science, and Technology of Japan (Kakenhi No: 20K08564) and in part by a grant from Shionogi & Co., Ltd.

## Conflict of Interest

ECG, CND-G, and TY have received a grant from Shionogi Co. to support in part the execution of the experimental study reported in the present manuscript, and SI and HS are employees from Shionogi & Co., Ltd. ECG and TK have a patent on the mouse used in this study.

The remaining authors declare that the research was conducted in the absence of any commercial or financial relationships that could be construed as a potential conflict of interest.
