# Supplementary material for: Low-Dose of Intrapulmonary Pirfenidone Improves Human Transforming Growth Factorβ1-Driven Lung Fibrosis
Source: Front Pharmacol. 2020 Nov 27;11:593620. doi: 10.3389/fphar.2020.593620 (PMC7774321; doi:10.3389/fphar.2020.593620)
Supplement: Supplementary file 1 [file datasheet1.pdf]

Online supplementary information

Low-dose of Intrapulmonary Pirfenidone Ameliorates Human Transforming Growth Factorβ1-driven Lung Fibrosis

Tomohito Okano, Tetsu Kobayashi, Taro Yasuma, Corina N. D'Alessandro-Gabazza, Masaaki Toda, Hajime Fujimoto, Hiroki Nakahara, Yuko Okano, Atsuro Takeshita, Kota Nishihama, Haruko Saiki, Atsushi Tomaru, Valeria Fridman D'Alessandro, Satoru Ishida, Hiromi Sugimoto, Yoshiyuki Takei, and Esteban C. Gabazza.

Supplementary Table 1. The dose of pirfenidone per group for intranasal administration

Group	Dose (mg/head)	Concentration (mg/mL)	Amount (μL/head)	Route of administration	Number of animals
WT/SAL	0	0	40	intranasal	7
TGFβ1-TG/SAL	0	0	40	intranasal	7
TGFβ1-TG/PFD0.04	0.04	1	40	intranasal	7
TGFβ1-TG/PFD 0.12	0.12	3	40	intranasal	7
TGFβ1-TG/PFD 0.4	0.4	10	40	intranasal	7

WT, wild type; TGFβ1, transforming growth factor-β1; SAL, saline; PFD, pirfenidone.

Supplementary Table 2. The dose of pirfenidone per group for oral administration

Group	Dose (mg/kg)	Concentration (mg/mL)	Amount (mL/kg)	Route of administration	Number of animals
WT/MC	0	0	10	oral	7
TGFβ1-TG/MC	0	0	10	oral	7
TGFβ1-TG/PFD-30	30	3	10	oral	7
TGFβ1-TG/PFD-100	100	10	10	oral	7
TGFβ1-TG/PFD-300	300	30	10	oral	7

WT, wild type; TGFβ1, transforming growth factor-β1; SAL, saline; PFD, pirfenidone.

Supplementary Table 3. The dose of pirfenidone per group for pharmacokinetics study after intranasal administration*

Group	Dose (mg/head)	Concentration (mg/mL)	Amount (μL/head)	Route of administration	Number of animals
TGFβ1-TG/PFD0.04	0.04	1	40	intranasal	4
TGFβ1-TG/PFD 0.12	0.12	3	40	intranasal	4
TGFβ1-TG/PFD 0.4	0.4	10	40	intranasal	4

*Pharmacokinetic study was conducted on day 1 and day 21 starting the experiment. WT, wild type; TGFβ1, transforming growth factor-β1; SAL, saline; PFD, pirfenidone.

Supplementary Table 4. The dose of pirfenidone per group for pharmacokinetic study after oral administration*

Group	Dose (mg/kg)	Concentration (mg/mL)	Amount (mL/kg)	Route of administration	Number of animals
TGFβ1-TG/PFD-30	30	3	10	oral	4
TGFβ1-TG/PFD-100	100	10	10	oral	4
TGFβ1-TG/PFD-300	300	30	10	oral	4

*Pharmacokinetic study was conducted on day 1 and day 21 starting the experiment. WT, wild type; TGFβ1, transforming growth factor-β1; SAL, saline; PFD, pirfenidone.

Supplementary Table 5. Primers for RT-PCR of mouse tissues

Gene		Sequence (5' to 3')	Length	Tm	Reference	Location	Product size
mouse TGFβ1	Sense	CTCCCGTGGCTTCTAGTGC	19	62.1	NM_011577	43-61	133 bp
	Antisense	GCCTTAGTTTGGACAGGATCTG	22	60.4		175-154	
human TGFβ1	Sense	CTAATGGTGGAAACCCACAACG	22	61.4	NM_000660	334-355	209 bp
	Antisense	TATCGCCAGGAATTGTTGCTG	21	60.7		542-522	
mouse IFNγ	Sense	ATGAACGCTACACACTGCATC	21	60.7	NM_008337	1-21	182 bp
	Antisense	CCATCCTTTTGCCAGTTCCTC	21	60.9		182-162	
mouse MCP-1	Sense	TAAAAACCTGGATCGGAACCAAA	23	64	NM_011333	261-283	120 bp
	Antisense	GCATTAGCTTCAGATTTACGGGT	23	66		380-358	
mouse IL-13	Sense	CCTGGCTCTTGCTTGCCTT	19	62.6	NM_008355	24-42	116 bp
	Antisense	GGTCTTGTGTGATGTTGCTCA	21	60.5		139-119	
mouse IL-6	Sense	CTGCAAGAGACTTCCATCCAG	21	60.1	NM_031168	14-34	131 bp
	Antisense	AGTGGTATAGACAGGTCTGTTGG	23	60.8		144-122	
mouse CTGF	Sense	GGGCCTCTTCTGCGATTTC	19	60.5	NM_010217	231-249	151 bp
	Antisense	ATCCAGGCAAGTGCATTGGTA	21	61.8		381-361	
mouse GAPDH	Sense	TGGCCTTCCGTGTTCCTAC	19	61.3	NM_008084	686-704	178 bp
	Antisense	GAGTTGCTGTTGAAGTCGCA	20	60.9		863-844	

TGFβ1: transforming growth factor-β1; SP-C, surfactant protein C; IFNγ, interferonγ; MCP-1, monocyte chemoattractant protein-1; IL-13, interleukin-13; IL-6, interleukin-6; CTGF, connective tissue growth factor; GAPDH, glyceraldehyde 3-phosphate dehydrogenase.

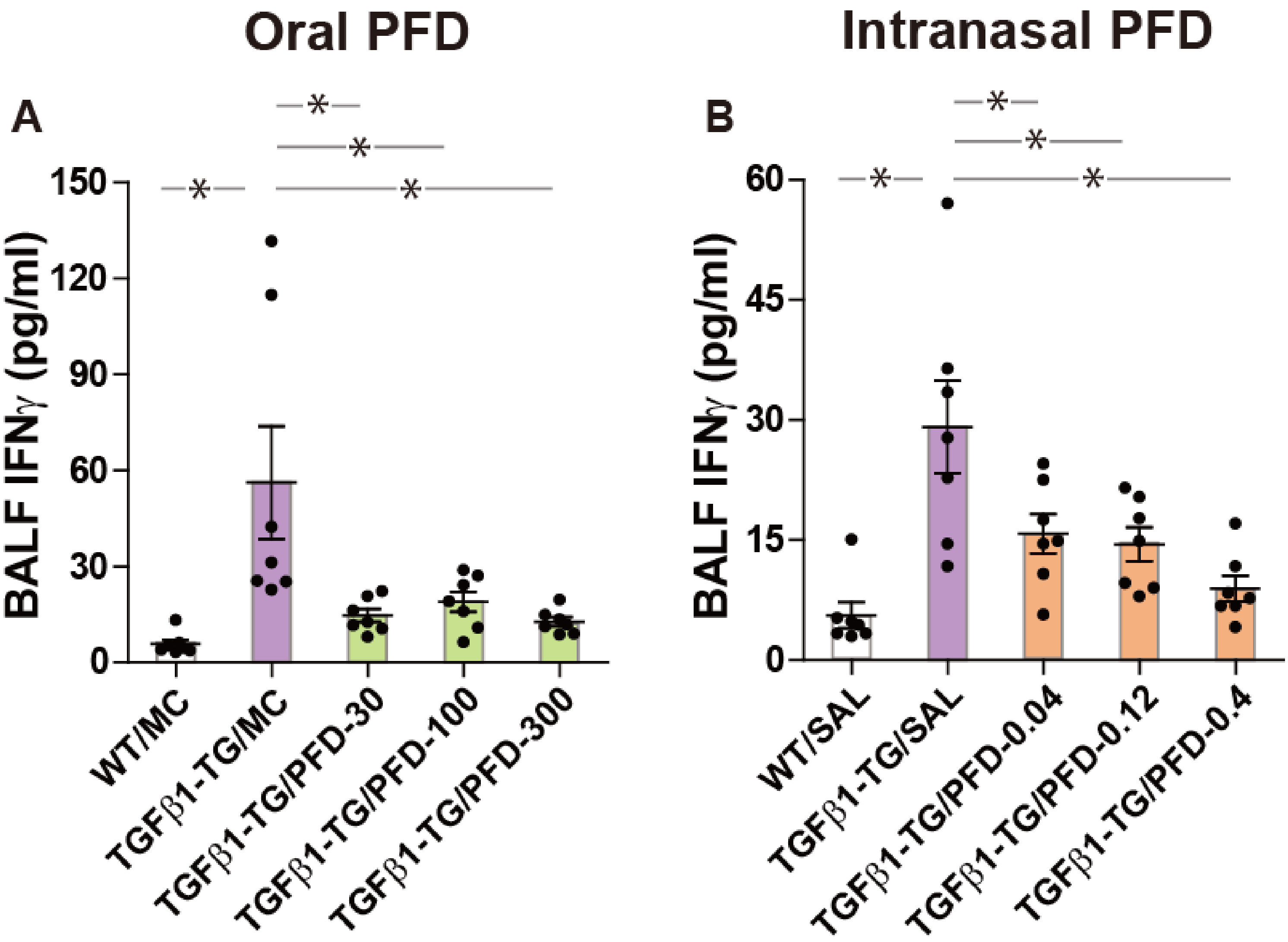

Supplementary Figure 1. Significant reduction in the concentration of interferon-γ in the bronchoalveolar lavage fluid from mice treated with pirfenidone (PFD) by oral or intranasal administration compared to controls. Human transforming growth factor (TGF)β1 transgenic (TG) mice were allocated in groups treated with PFD by oral (**a**; n=7) or intranasal (**b**; n=7) administration and in groups treated with the vehicle saline (SAL; n=7) or with the vehicle methylcellulose (MC; n=7) twice a day for 21 days and once a day on day 22 before euthanasia. Wild type (WT; n=7) mice treated with SAL or MC were used as negative controls. The concentration of interferonγ (IFNγ) was measured by enzyme immunoassays as described under materials and methods. Data are expressed as the mean ± S.E.M. Statistical analysis by Student's t test and ANOVA with Dunnett's *post hoc* test. *$p<0.05$.

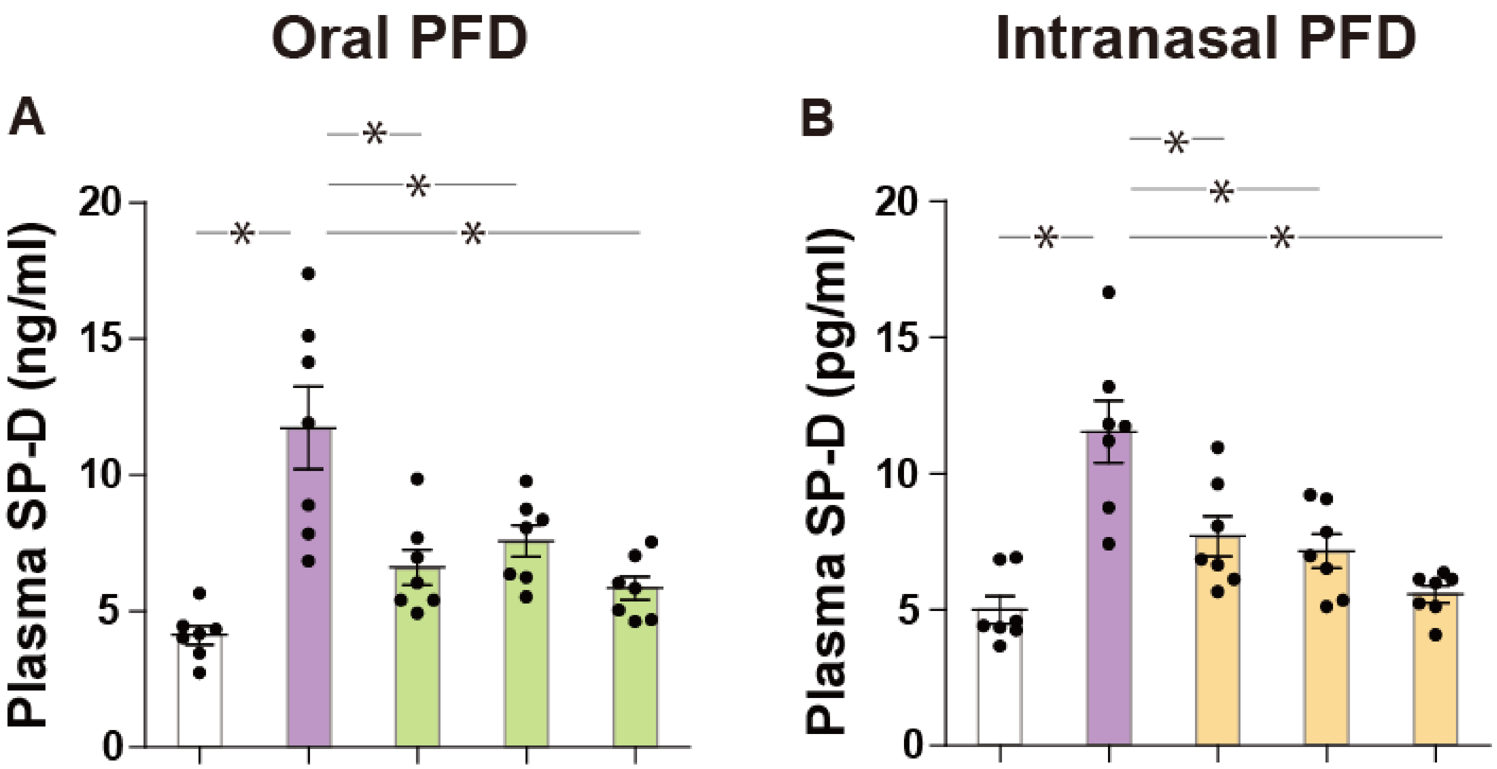

Supplementary Figure 2. Significant reduction in the circulating level of surfactant protein D (SP-D) in the lungs from mice treated with pirfenidone (PFD) by oral or intranasal administration compared to controls. Human transforming growth factor (TGF)β1 transgenic (TG) mice were allocated in groups treated with PFD by oral (**a**; n=7) or intranasal (**b**; n=7) administration and in groups treated with the vehicle saline (SAL; n=7) or with the vehicle methylcellulose (MC; n=7) twice a day for 21 days and once a day on day 22 before euthanasia. Wild type (WT; n=7) mice treated with SAL or MC were used as negative controls. The concentration of SP-D was measured by enzyme immunoassay as described under materials and methods. Data are expressed as the mean ± S.E.M. Statistical analysis by Student's t test and ANOVA with Dunnett's *post hoc* test. *$p<0.05$.

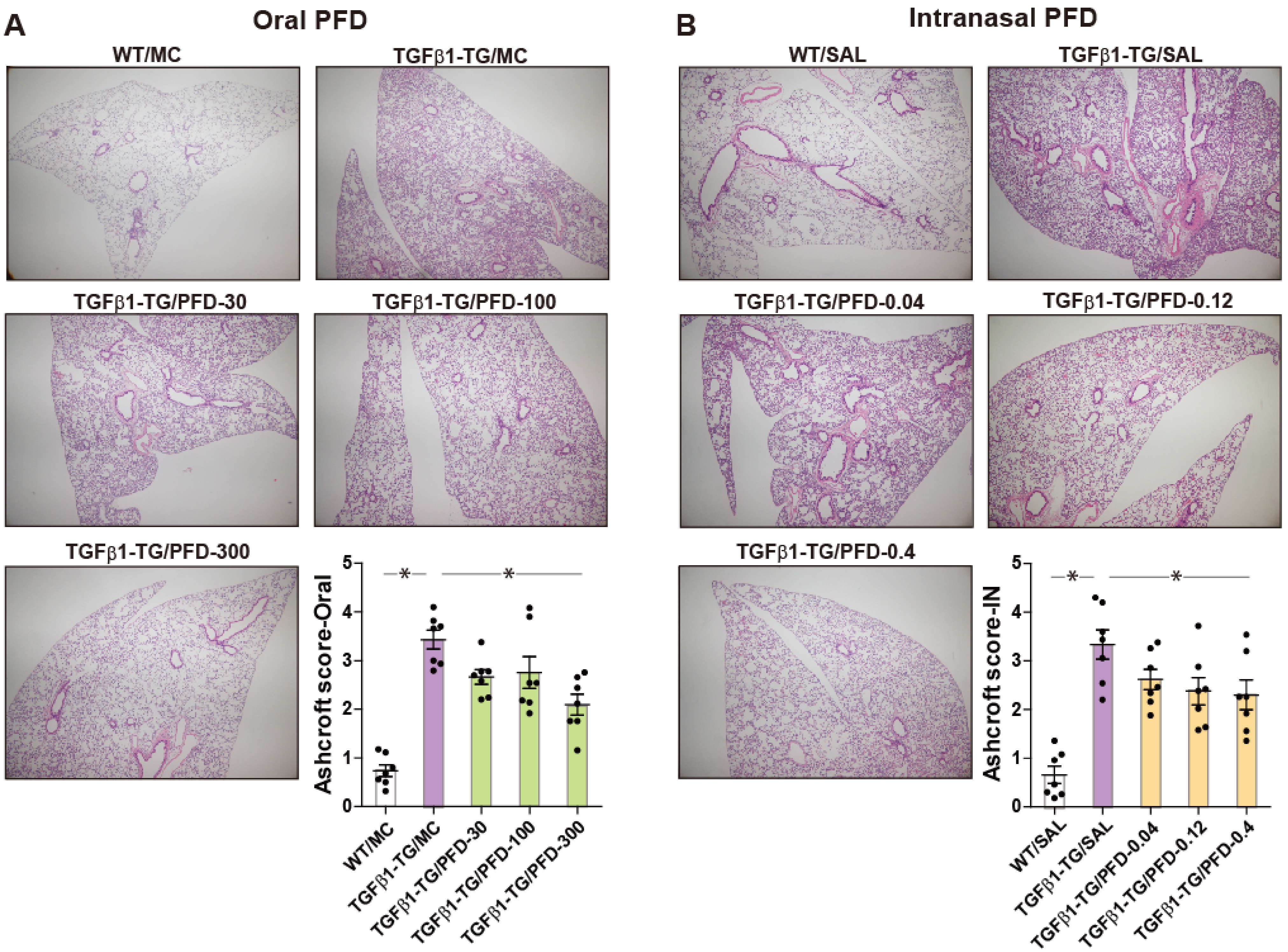
A
Oral PFD
WT/MC
TGFβ1-TG/MC
TGFβ1-TG/PFD-30
TGFβ1-TG/PFD-100
TGFβ1-TG/PFD-300
Ashcroft score-Oral
WT/MC
TGFβ1-TG/MC
TGFβ1-TG/PFD-30
TGFβ1-TG/PFD-100
TGFβ1-TG/PFD-300
B
Intranasal PFD
WT/SAL
TGFβ1-TG/SAL
TGFβ1-TG/PFD-0.04
TGFβ1-TG/PFD-0.12
TGFβ1-TG/PFD-0.4
Ashcroft score-IN
WT/SAL
TGFβ1-TG/SAL
TGFβ1-TG/PFD-0.04
TGFβ1-TG/PFD-0.12
TGFβ1-TG/PFD-0.4

Supplementary Figure 3. Significant reduction in the Ashcroft lung fibrosis score after treatment with oral or intranasal pirfenidone (PFD) compared to controls. Human transforming growth factor (TGF)β1 transgenic (TG) mice were allocated in groups treated with PFD by oral (**a**; n=7) or intranasal (**b**; n=7) administration and in groups treated with the vehicle saline (SAL; n=7) or with the vehicle methylcellulose (MC; n=7) twice a day for 21 days and once a day on day 22 before euthanasia. Wild type (WT; n=7) mice treated with saline or MC were used as negative controls. Lung tissue was stained with hematoxylin & eosin, and fibrosis scoring was performed by nine readers who were blinded to the treatment and mouse groups as described under materials and methods. Scale bars indicate 500 μm. Data are expressed as the mean ± S.E.M. Statistical analysis by Student's t-test and ANOVA with Dunnett's *post hoc* test. *$p<0.05$.

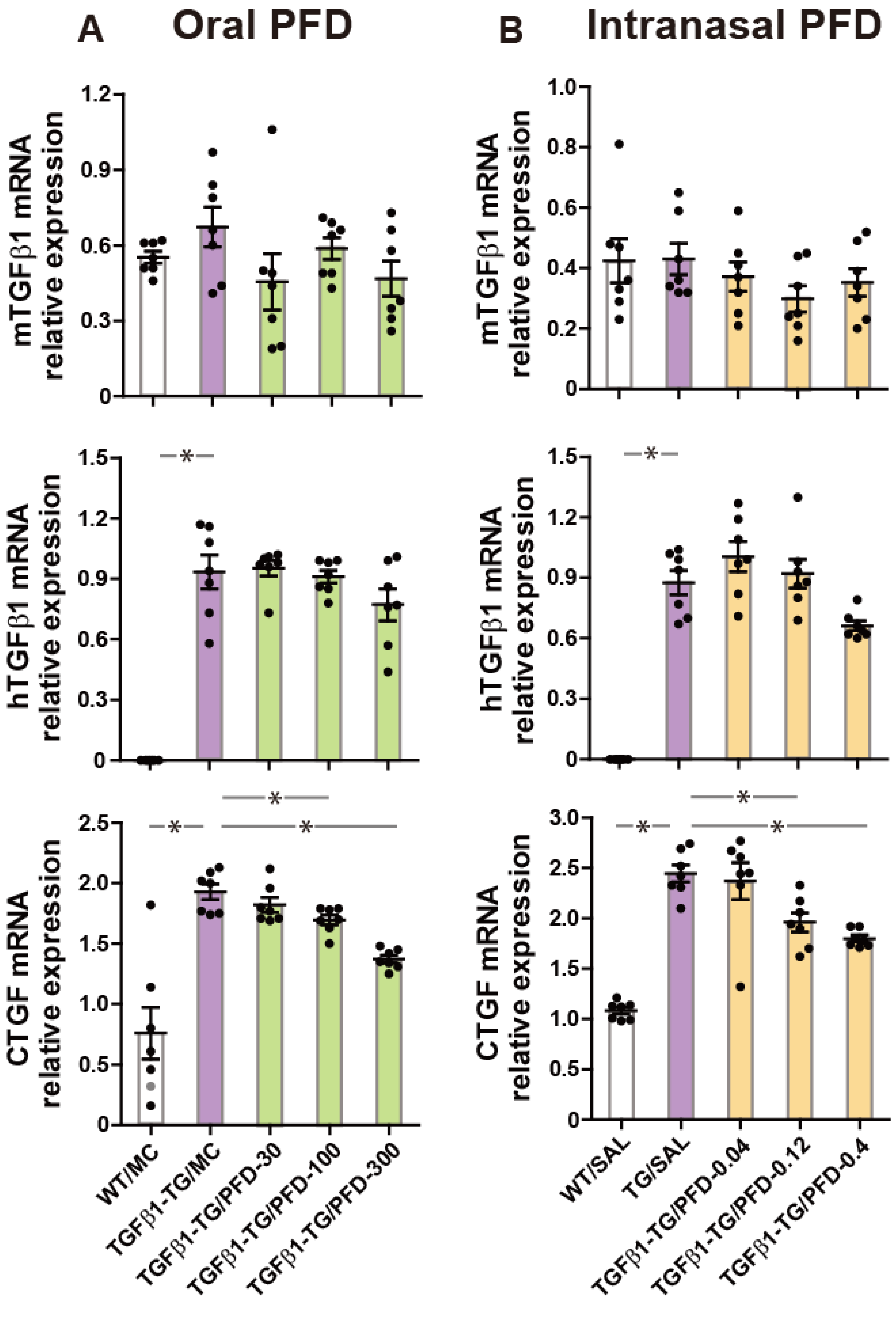

A
Oral PFD
B
Intranasal PFD
mTGFβ1 mRNA relative expression
hTGFβ1 mRNA relative expression
CTGF mRNA relative expression
*
WT/MC
TGFβ1-TG/MC
TGFβ1-TG/PFD-30
TGFβ1-TG/PFD-100
TGFβ1-TG/PFD-300
WT/SAL
TG/SAL
TGFβ1-TG/PFD-0.04
TGFβ1-TG/PFD-0.12
TGFβ1-TG/PFD-0.4

Supplementary Figure 4. Significant reduction in the relative mRNA expression of pro-fibrotic growth factors in the lungs from mice treated with pirfenidone (PFD) by oral or intranasal administration compared to controls. Human transforming growth factor (TGF)β1 transgenic (TG) mice were allocated in groups treated with PFD by oral (**a**; n=7) or intranasal (**b**; n=7) administration and in groups treated with the vehicle saline (SAL; n=7) or with the vehicle methylcellulose (MC; n=7) twice a day for 21 days and once a day on day 22 before euthanasia. Wild type (WT; n=7) mice treated with SAL or MC were used as negative controls. The relative mRNA expression of mouse (m) and human (h) TGFβ1 and connective tissue growth factor (CTGF) was evaluated by RT-PCR as described under materials and methods. Data are expressed as the mean ± S.E.M. Statistical analysis by Student's t test and ANOVA with Dunnett's *post hoc* test. *p<0.05.

Supplementary Table 6. Pharmacokinetic variables after the first administration of PFD on day 1 and after repeated administrations of PFD on day 22

PK parameters	Oral: 30 mg/kg Intranasal: 0.04 mg/head		Oral: 100 mg/kg Intranasal: 0.12 mg/head		Oral: 300 mg/kg Intranasal: 0.4 mg/head	
	Day 1	Day 22	Day 1	Day 22	Day 1	Day 22
**Cmax (ng/mL)**						
Oral	10600 ± 2700	16700 ± 5600	49600 ± 27800	27500 ± 6200	134000 ± 18000	90400 ± 9900*
intranasal	531 ± 71	548 ± 76	1630 ± 70	1700 ± 290	6560 ± 1100	6040 ± 1920
**Tmax (h)**						
Oral	0.146 ± 0.042	0.146 ± 0.042	0.229 ± 0.185	0.146 ± 0.042	0.229 ± 0.185	0.146 ± 0.042
intranasal	0.125 ± 0.048	0.146 ± 0.042	0.146 ± 0.042	0.125 ± 0.048	0.146 ± 0.042	0.104 ± 0.042
**t1/2,z (h)**						
Oral	1.61 ± 0.38	2.20 ± 0.94	1.23 ± 0.60	2.29 ± 0.80	1.24 ± 0.19	2.10 ± 0.37*
intranasal	0.605 ±0.291	0.392 ± 0.055	0.630 ± 0.062	0.375 ± 0.047*	0.487 ±0.087	0.274 ± 0.024*
**AUCinf (ng·h/mL)**						
Oral	20500 ± 6800	23600 ± 10000	75200 ± 34000	35900 ± 2700	297000 ± 47000	129000 ± 84000*
intranasal	483 ± 134	385 ± 33	1670 ± 150	1120 ± 140*	4430 ± 290	3200 ± 1020

Data are expressed as the mean ± S.D. of 4 mice. Cmax, maximum concentration; Tmax, time to reach the maximum concentration; t1/2,z, elimination half-life; AUCinf, the area under the concentration-time curve from zero to infinity time; AUClast, area under the plasma concentration-time curve from time zero to the last quantifiable time; h, hours. Statistical analysis by t-test with Welch's correction.
